# β‐Sitosterol as an Anti‐Tumour Active Component of *Herba Sarcandrae* Inhibits Colorectal Cancer Progression Through Up‐Regulation of TBX20

**DOI:** 10.1111/jcmm.70809

**Published:** 2025-09-01

**Authors:** Haixiao Yuan, Weiqing Feng, Shaohua Yang, Hao Yin, Shaoyong Ouyang, Hong Xie, Hongmei Tang, Xiaowei Ou, Xianling Gong, Jie Yuan

**Affiliations:** ^1^ Director's Office Guancun Health Center Guangzhou Guangdong China; ^2^ Foshan Clinical Medical School Guangzhou University of Chinese Medicine Foshan Guangdong China; ^3^ Department of General Surgery Foshan Fosun Chancheng Hospital Foshan Guangdong China; ^4^ Pharmaceutical Department First Affiliated Hospital of Guangzhou University of Chinese Medicine Guangzhou Guangdong China; ^5^ School of Pharmacy Guangdong Medical University Dongguan Guangdong China; ^6^ The Affiliated Fosun Chancheng Hospital Guangdong Medical University (Foshan Fosun Chancheng Hospital) Foshan Guangdong China

**Keywords:** β‐Sitosterol, colorectal cancer, *Herba Sarcandrae*, network pharmacology, TBX20

## Abstract

Colorectal cancer (CRC) is a common malignant tumor of the digestive tract with a high incidence rate. *Herba Sarcandrae* (HS) is an antipyretic and has been reported to have anti‐cancer effects. This study explored the impacts of β‐sitosterol on the sensitivity of CRC to 5‐fluorouracil (5‐FU) and oxaliplatin and the stability of TBX20 protein in CRC cells. There were 41 HS active ingredients and 265 corresponding potential targets, and 48 potential targets of HS were enriched in CRC. Then, 206 differentially expressed genes (DEGs) related to TBX20 overexpression were screened based on the TCGA database, some of which were associated with TMN stages of COAD patients. Epimedin C, rutin, and β‐sitosterol, which could be combined with TBX20, were screened and validated in CRC cells. Functionally, β‐sitosterol could suppress proliferation and induce apoptosis of CRC cells. β‐sitosterol could also enhance the sensitivity of CRC to 5‐FU and oxaliplatin. In xenograft models, both HS and β‐sitosterol treatments inhibited tumor growth and upregulated TBX20 protein expression, with β‐sitosterol demonstrating a stronger effect. Mechanistically, β‐sitosterol may stabilize TBX20 by inhibiting its ubiquitin‐mediated degradation. In conclusion, β‐sitosterol, as an anti‐tumor active component of HS, prevents CRC cell proliferation, and accelerates apoptosis by upregulating TBX20.

**Abbreviations**
5‐FU: 5‐fluorouracil
CRC: Colorectal cancer
DEGs: differentially expressed genes
HS: *Herba Sarcandrae*
IHC: immunohistochemistry
MCODE: molecular complex detection
OD: optical density
PI: propidium iodide
PPI: protein–protein interaction
SPF: specified pathogen‐free
TCM: Traditional Chinese medicine

## 1 Introduction

*Herba Sarcandrae* (HS) is the dried whole plant of Sarcandra glabra (Thunb.) Nakai of the Chrysanthemum family [1]. HS is rich in coumarin, flavonoids, organic acids, and other functional components, which can play the role of dispelling wind and cold, promoting blood circulation, eliminating spots, clearing heat, and cooling blood [2]. Studies proved that HS has multiple effects, such as anti‐inflammatory, analgesic, anti‐tumor, antibacterial, immune regulation, liver protection, and anti‐oxidation [2, 3]. In recent years, studies have confirmed that HS extracts suppress malignant tumor progression mainly by inhibiting cell proliferation, telomerase activity, invasion and migration, and inducing cell apoptosis [3, 4]. However, the specific targets and molecular mechanisms of HS active components are still unclear.

Drugs developed under the traditional concept of “single drug‐single target‐single disease” have been characterized by multiple side effects and poor efficacy in the treatment of complex diseases [5]. Traditional Chinese medicine (TCM) is characterized by the synergistic effect of “multi‐components, multi‐targets and multi‐pathways”, so it has unique advantages in the treatment of complex diseases [6]. Network pharmacology is an emerging discipline that integrates systems biology, bioinformatics, and multi‐directional pharmacology [5]. Based on the “disease‐gene‐target‐drug” interaction network, it the shift from “single target” to “network target” research mode, which is consistent with the holistic view of TCM and the action mode of TCM system regulation [7]. Therefore, network pharmacology provides a new idea and method for the research and development of new drugs.

T‐box transcription factor plays different roles during embryonic development [8]. TBX20 is one of the crucial members of the T‐box gene family [9]. TBX20 is located on chromosome 7 (7p14.2) and has eight exons, which can regulate transcription by binding to specific DNA sequences [10]. Previous study and our unpublished result demonstrated that TBX20 is a key tumor suppressor gene in colorectal cancer [11]. Whether there is an active component in HS targeting TBX20 to inhibit CRC remains to be investigated.

In this study, the network pharmacology method was applied to construct and analyze the HS component‐target‐disease network, and the key components, targets, and disease types were extracted. The material basis and molecular mechanism of HS were explored at the level of system biology. By comprehensive analysis of TBX20 target genes and HS target genes, the active components and target genes of HS were obtained, which were related to TBX20 target genes and CRC. Functionally, the present study further investigated the effects of the active components of HS that can target TBX20 on CRC progression. This will provide a reference for CRC therapy.

## 2 Materials and Methods

### 2.1 Acquisition and Analysis of Active Ingredients of HS

Relevant targets of the active components of HS were obtained using multiple drug‐related databases including SymMap [12], TCMID [13], TCMSP [14], and TCM‐ID [15]. The major active ingredient β‐sitosterol was further validated using PubChem (https://pubchem.ncbi.nlm.nih.gov) with Compound ID: 482022047. The UniProt database (https://www.uniprot.org/) was applied to correct the matching target gene names. The target network of HS and the active ingredient was constructed using Cytoscape 3.7.2 software. The potential targets of HS were identified by Disease Ontology (DO) analysis [16], Gene Ontology (GO) analysis, and KEGG analysis [17]. Raw molecular docking results and PDB files are provided in Table S1.

### 2.2 TCGA Data Analysis

Then the transcriptome expression data related to CRC was downloaded using the TCGA database (https://portal.gdc.cancer.gov/). The COAD dataset (Colon Adenocarcinoma, Project ID: TCGA‐COAD) was applied for subsequent analysis. The clinical data was organized and summarized using R software, and TBX20 overexpression‐related genes were further obtained. Based on the screening criteria of *p* Value < 0.05 and FDR ≥ 1.5‐fold (|log2 FC| ≥ 0.585), heatmap and volcano diagram of TBX20 overexpression‐related genes were visualized using R language. Besides, the prognostic significance of TBX20 expression was evaluated with Kaplan–Meier survival analysis in COAD.

### 2.3 Protein–Protein Interaction (PPI) Network

The PPI network was confirmed using the STRING database (https://string‐db.org/) to analyze DEGs mediated by TBX20.

### 2.4 Molecular Complex Detection (MCODE) Analysis

The target network of HS targets‐TBX20 overexpressed DEGs‐WGCNA was constructed using Cytoscape 3.7.2 software. The potential core target genes of HS targets combined with TBX20 for treating CRC patients were predicted using MCODE based on the Degree algorithm [18].

### 2.5 Cell Culture

HCT‐116 and HT‐29 cells were from Shanghai Cell Bank, Chinese Academy of Sciences, and incubated in DMEM, F12 (Gibco, Grand Island, NY, USA) with 10% FBS (Gibco, Rockville, MD, USA) at 37°C with 5% CO_2_.

### 2.6 Cell Treatment

HCT‐116 and HT‐29 cells were processed with 0, 10, 50, 100, and 500 μM Epimedin C, rutin, and β‐sitosterol for 48 h, respectively. HCT‐116 and HT‐29 cells were also processed with 50 μM β‐sitosterol, 5 μg/mL 5‐fluorouracil (5‐FU), and 5 μg/mL Oxaliplatin for 48 h. The IC50 values of each compound were determined based on the CCK‐8 assay results. TBX20 siRNA‐1, TBX20 siRNA‐2, and negative control (NC) were obtained from Genepharma (Shanghai, China). HCT‐116 and HT‐29 cells were transfected with NC, TBX20 siRNA‐1, and TBX20 siRNA‐2 using LipofectamineTM 3000 (Invitrogen) for 48 h based on the kit instructions.

### 2.7 CCK‐8

HCT‐116 and HT‐29 cells (1 × 10^4^ cells/well) were seeded in 96‐well plates and cultured overnight at 37°C in a 5% CO_2_ incubator. At 24, 48, and 72 h, each well was supplemented with 10 μL CCK‐8 (Invitrogen), mixed, and cultured in the incubator for another 3 h. The optical density (OD) value was monitored at 450 nm by an automatic microplate reader.

### 2.8 Flow Cytometry

The processed cells were harvested and resuspended with 100 μL binding buffer and adjusted to a density of 5 × 10^5^ cells/mL. After treatment with Annexin V and PI (BD Biosciences, Franklin Lakes, NJ, USA) for 15 min, apoptosis was analyzed by flow cytometry.

### 2.9 Animal

A total of 18 male nude mice (4–6 weeks, weighing: 18–22 g) were used in this study. All mice were housed under specified pathogen‐free (SPF) conditions at a room temperature of 26°C–28°C, a humidity of 40%–60%, and were fed regularly with autoclaved drinking water and chow. All animal experiments were approved by the Ethical Committee of Guangzhou Forevergen Medical Laboratory Animal Center (approval no: IACUC‐AEWC‐F2211008). All animal studies should also comply with the ARRIVE guidelines and the AVMA euthanasia guidelines 2020.

### 2.10 Establishment of Subcutaneous Xenotransplant Tumour Model in Nude Mice

The nude mice were randomly divided into the control group, β‐sitosterol group, and HS group, with 6 mice in each group. HT‐29 cells (0.5 mL, 1 × 10^7^ cells/mL) were subcutaneously inoculated into the right axillae of nude mice. After 14 days, when tumors became palpable, drug treatment was initiated. HS was administered by oral gavage at 0.2 mL (6.24 g/kg) once daily, and β‐sitosterol was delivered by intraperitoneal injection at the same volume (50 mg/kg) and frequency. The control group received an equivalent volume of vehicle. Treatment lasted for 12 consecutive days. At the end of the experiment, mice were euthanized under isoflurane anesthesia. Tumor tissues were collected, with one portion fixed in 10% formaldehyde for H&E staining and immunohistochemistry (IHC), and the remaining tissue snap‐frozen in liquid nitrogen for further analysis.

### 2.11 H&E Staining

The tumor sections were dewaxed using xylene and dehydrated using gradient alcohol. Then, the sections were treated with hematoxylin (Solarbio, Beijing, China) for 5 min, 1% hydrochloric acid (Sigma‐Aldrich, Chemical Co, USA), water rinsing, and eosin (Sigma‐Aldrich, USA) for 3 min. After dehydration, transparency, and sealing, the pathological structure was photographed with a light microscope (CCD TP510, Optec Inc., Lowell, MI, USA).

### 2.12 IHC Assay

The sections were deparaffinized and dehydrated using the gradient ethanol and treated with 3% H_2_O_2_ for 10 min. Then, antigen retrieval was conducted using 0.01 M sodium citrate. After blocking with the goat serum, the sections were incubated with the Ki67 antibody (1: 100; Abcam) at 4°C overnight. After washing, the sections were processed with HRP‐labeled secondary antibody (1: 200; Abcam) at 37°C for 30 min. After washing, the sections were stained with 3.3′ diaminobenzidine (DAB, Dako, Glostrup, Denmark), counterstained with hematoxylin (OriGene Technologies Inc., Rockville, MD, USA), differentiated, reverse blue, hydrated, transparent, and sealed with neutral gum. The results were observed under a microscope (×200).

### 2.13 CRC Organoid Culture

The CRC organoid medium (Biorgen, China) was prepared using Advanced DMEM/F12 (Invitrogen), A83‐01 (500 nM, Tocris), Noggin (10%, PrimeGene, Shanghai, China), SB202190 (10 μM, ApexBio), R‐Spondin 1 (10%, Absin, Shanghai, China), Prostaglandin E2 (5 μg/mL, Cayman Chemical, Ann Arbor, MI, USA), gastrin I (1 nmol/L, Tocris Bioscience), B27 (2%, Invitrogen), Y‐27632 (10 mmol/L, Sigma‐Aldrich), N‐Acetylcysteine (1.25 mM, Sigma‐Aldrich), epidermal growth factor (50 ng/mL, Invitrogen), and Nicotinamide (10 μM, Sigma‐Aldrich). The extracted CRC tissues were digested using the digestive solution (Biorgen) for 2 h and suspended using CRC organoid medium (Biorgen), which was supplemented with Matrigel (Xiamen Mogengel, China) in a 1:1 ratio. 50 μL of the mixture was added to a 48‐well plate at 37°C for 5 min and allowed to solidify for 20 min. After coagulation, 200 μL of CRC organoid medium was added to 48‐well culture plates for culture. The growth of organoids was observed, and the culture medium was replaced in time.

### 2.14 Organoids ATP Activity Assay

The organoids were inoculated in 96‐well plates and cultured at 37°C with 5% CO_2_. When the average diameter was > 50 μm, the medium was changed. The organoids were treated with 50 μM β‐sitosterol, 5 μg/mL 5‐FU, 5 μg/mL Oxaliplatin, 50 μM β‐sitosterol plus 5 μg/mL 5‐FU, and 50 μM β‐sitosterol plus 5 μg/mL Oxaliplatin for 48 h. The organoids were left at room temperature for 30 min. Cell Counting‐Lite 3D (Vazyme) was added with 100 μL cell culture. After complete dissolution and stabilization, the ATP activity of the organoid was assayed with an ATP kit.

### 2.15 qRT‐PCR

Total RNA was extracted using TRIzol (Invitrogen, MA, USA). After purity and concentration detection, total RNA was reverse transcribed into cDNA using the reverse transcription kit (Takara, Tokyo, Japan). TBX20 mRNA expression was measured by qPCR using SYBR Green qPCR Master Mix (DBI Bioscience) with cDNA as a template. The expression of TBX20 was quantified using the 2^−ΔΔCt^ method.

### 2.16 Western Blot

Groups of cells were added with pre‐cooled RIPA (Beyotime, Ningbo, China). After quantification, 40 μg of protein was separated by 10% SDS‐PAGE and transferred to the PVDF membrane (Millipore, Bedford, MA, USA). The samples were closed and incubated with TBX20 antibody (1: 1000; Abcam, Cambridge, MA, USA) overnight at 4°C and goat anti‐rabbit IgG (1: 2000; Abcam, Cambridge, MA, USA) for 1 h. After washing, the samples were exposed by dropwise addition of ECL for color development.

### 2.17 Protein Thermal Shift Technology

HCT‐116 cells (2 × 10^7^) were digested with trypsin and harvested through centrifugation. The HCT‐116 cells were washed with PBS and resuspended in 2 mL of PBS supplemented with a complete protease inhibitor. Cell suspensions were repeatedly freeze‐thawed three times in liquid nitrogen and 37°C water to lyse the cells. The soluble fraction (cell lysate) was separated from the cellular debris by centrifugation at 20,000 × g for 20 min at 4°C. The supernatant was divided into two groups: the 100 μM β‐sitosterol‐treated group and the control group (equal volume of DMSO). The samples were incubated at 37°C for 30 min. The lysates were divided into smaller aliquots (80 μL/group) and placed at 37°C, 40°C, 43°C, 46°C, 52°C, 56°C, 60°C, and 63°C for 3 min. After 3 min at room temperature, the samples were placed at 4°C for 3 min after centrifugation at 20,000 × g for 20 min. The supernatant was boiled at 100°C for 5 min and added to the supersampling buffer. Finally, a Western blot assay was performed. Based on the above experiments, the appropriate temperature (55°C) was determined. After repeated freezing and thawing, the cell lysates were divided into 4 groups: 0 μM, 10 μM, 50 μM, and 100 μM β‐sitosterol‐treated groups. The other steps are the same as above.

### 2.18 Co‐Immunoprecipitation (CO‐IP) Assay

HCT‐116 and HT‐29 cells were treated with 100 μM β‐sitosterol or DMSO for 48 h. Cells were lysed in cold RIPA buffer with protease inhibitors, followed by centrifugation to remove debris. The supernatant was incubated with TBX20 antibody overnight at 4°C. Protein A/G agarose beads were used to capture the TBX20‐antibody complexes. After washing, proteins were eluted, and TBX20 ubiquitination was assessed using a ubiquitin‐specific antibody via Western blotting. TBX20 expression was also analyzed to verify the results.

### 2.19 Statistical Analysis

Results are expressed as mean ± SD from three independent experiments. SPSS 21.0 software was applied for statistical analysis with a one‐way analysis of variance. *p* < 0.05 was considered statistically significant.

## 3 Results

### 3.1 Cyberpharmacological Analysis of the Components and Targets of HS

The relevant targets of the active ingredients of HS were obtained by searching several drug‐related databases, including SymMap, TCMID, TCMSP, and TCM‐ID. After Uniprot database correction, 41 HS active ingredients and 265 corresponding potential targets were obtained. Then, the HS‐active ingredient‐target network was constructed through Cytoscape 3.7.2 software (Figure S1A). DO analysis showed that 48 potential targets of HS were enriched in CRC, including PTGS2, DPP4, MAPK14, GSK3B, ACHE, CCND1, TNF, BAX, BCL2, CASP3, and CASP8 (Figure S1B). GO analysis showed that the potential targets of HS were mainly enriched in response to xenobiotic stimulus, response to nutrient levels, response to lipopolysaccharide, response to molecules of bacterial origin, response to oxygen levels, etc. (Figure S1C). KEGG analysis denoted that the potential targets of HS were mainly enriched in AGE‐RAGE signaling pathway in diabetic complications, lipid and atherosclerosis, fluid shear stress and atherosclerosis, etc. (Figure S1D).

### 3.2 Association of TBX20 and Its Target Genes With CRC

**FIGURE 1:**
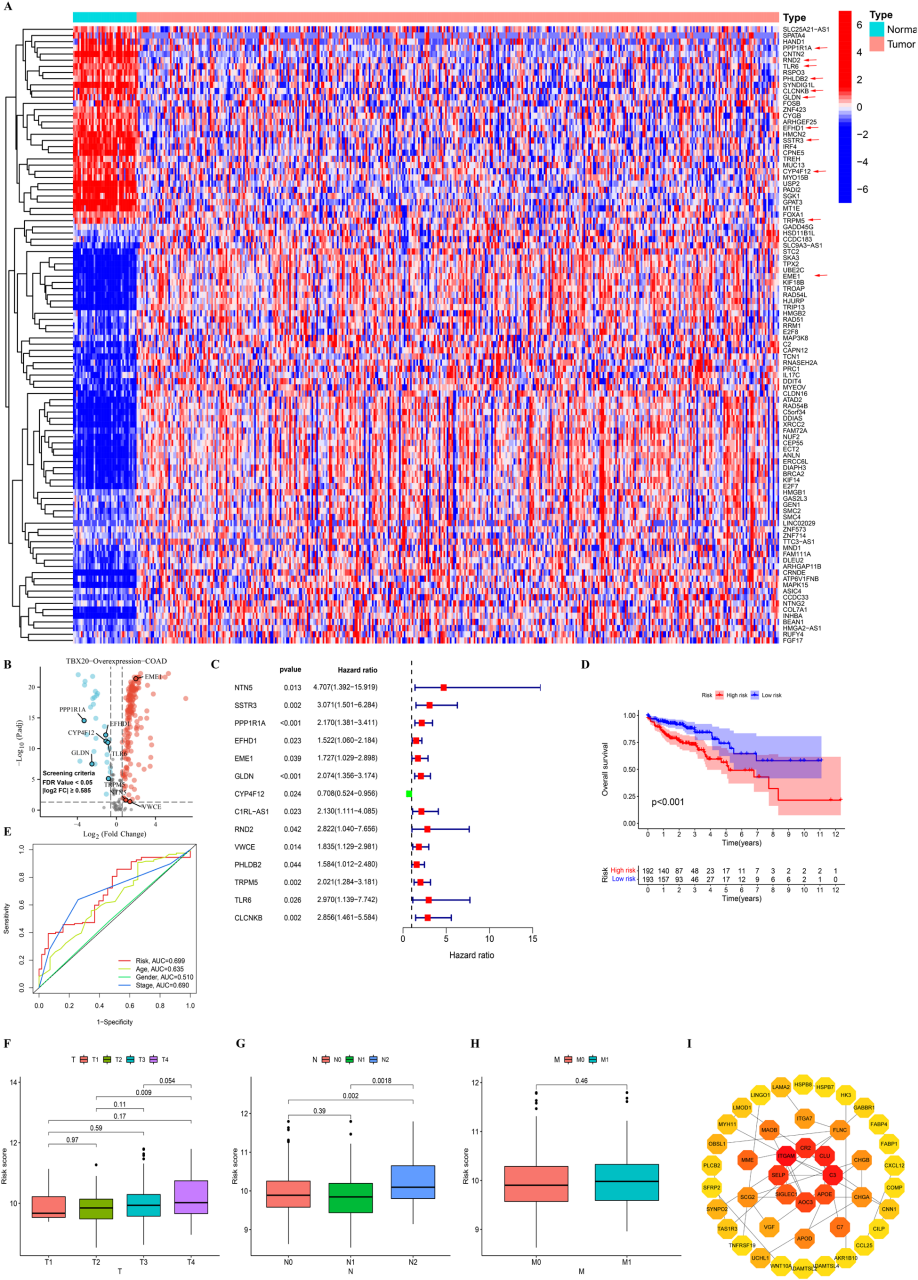
Association of TBX20 and its target genes with CRC. Screening of DEGs in colon adenocarcinoma (COAD) with TBX20 overexpression using the TCGA database. (A) Heatmap of expression of TBX20 overexpression‐related genes, with arrows indicating key genes of interest. (B) Volcano diagram of TBX20 overexpression‐related genes. Genes were selected based on a *p* value threshold of < 0.05 and a fold change greater than 1.5 (|log2 FC| ≥ 0.585). Red represents up‐regulated genes and blue represents down‐regulated genes. (C) Survival analysis of NTN5, SSTR3, PPP1R1A, EFHD1, EME1, GLDN, CYP4F12, C1RL‐AS1, RND2, VWCE, PHLDB2, TRPM5, TLR6, and CLCNKB in the TCGA database and clinical data of COAD. (D) The survival prognosis analysis of COAD‐TCGA patients with the prognostic models constructed by NTN5, PPP1R1A, EFHD1, EME1, GLDN, CYP4F12, C1RL.AS1, VWCE, TRPM5, and TLR6. (E) ROC curve analysis of TBX20 overexpressed risk genes in CRC. Effect of COAD‐TBX20 overexpression on different TMN stages, including *T*‐stage (F), *N*‐stage (G), and *M*‐stage (H), of tumors from COAD patients. (I) PPI analysis of DEGs mediated by TBX20.

Through the TCGA database, 206 DEGs were screened in the dataset, including 174 up‐regulated genes and 32 down‐regulated genes. The expression profile of TBX20 overexpression‐related DEGs was displayed using a Heatmap (Figure 1A) and Volcano diagram (Figure 1B). Then, a one‐way analysis of data presented that COAD survival prognosis was associated with high expression of NTN5, EME1, C1RL‐AS1, and VWCE and low expression of SSTR3, PPP1R1A, EFHD1, GLDN, CYP4F12, RND2, PHLDB2, TRPM5, TLR6, and CLCNKB (Figure 1C). COAD survival prognosis‐associated genes were analyzed with GEO‐COAD clinical data for survival prognosis. The data indicated that NTN5, PPP1R1A, EFHD1, EME1, GLDN, CYP4F12, C1RL.AS1, VWCE, TRPM5, and TLR6 expression had a significant effect on survival prognosis in COAD (Figure S2A–J). A prognostic model was constructed with the above 10 genes. The results showed that the model was significant for the survival prognosis of COAD‐TCGA patients (Figure 1D). The ROC curve of CRC risk factors showed that the area under the curve (AUC) of TBX20 overexpression risk genes was the largest, which was 0.699, and the AUC of age, gender, and stage were 0.635, 0.510, and 0.690, respectively (Figure 1E). The analysis data of the COAD‐TBX20 overexpression prognostic model revealed that TBX20 overexpression risk genes had different degrees of influence on different TMN stages of COAD patients (Figure 1F–H). Subsequently, TBX20 DEGs were subjected to PPI and topology analysis, and their network diagrams are shown in Figure 1I. Kaplan–Meier survival analysis of TBX20 expression in COAD patients revealed that high TBX20 expression was associated with poorer survival outcomes (*p* = 0.0099) (Figure S3). The results showed that genes such as ITGAM, CR, CLU, C3, APOE, AOC3, SELP, and SILEC1 were located at the core.

### 3.3 Correlation Between the Targets of Active Components in HS and the Core Target Genes of TBX20

**FIGURE 2:**
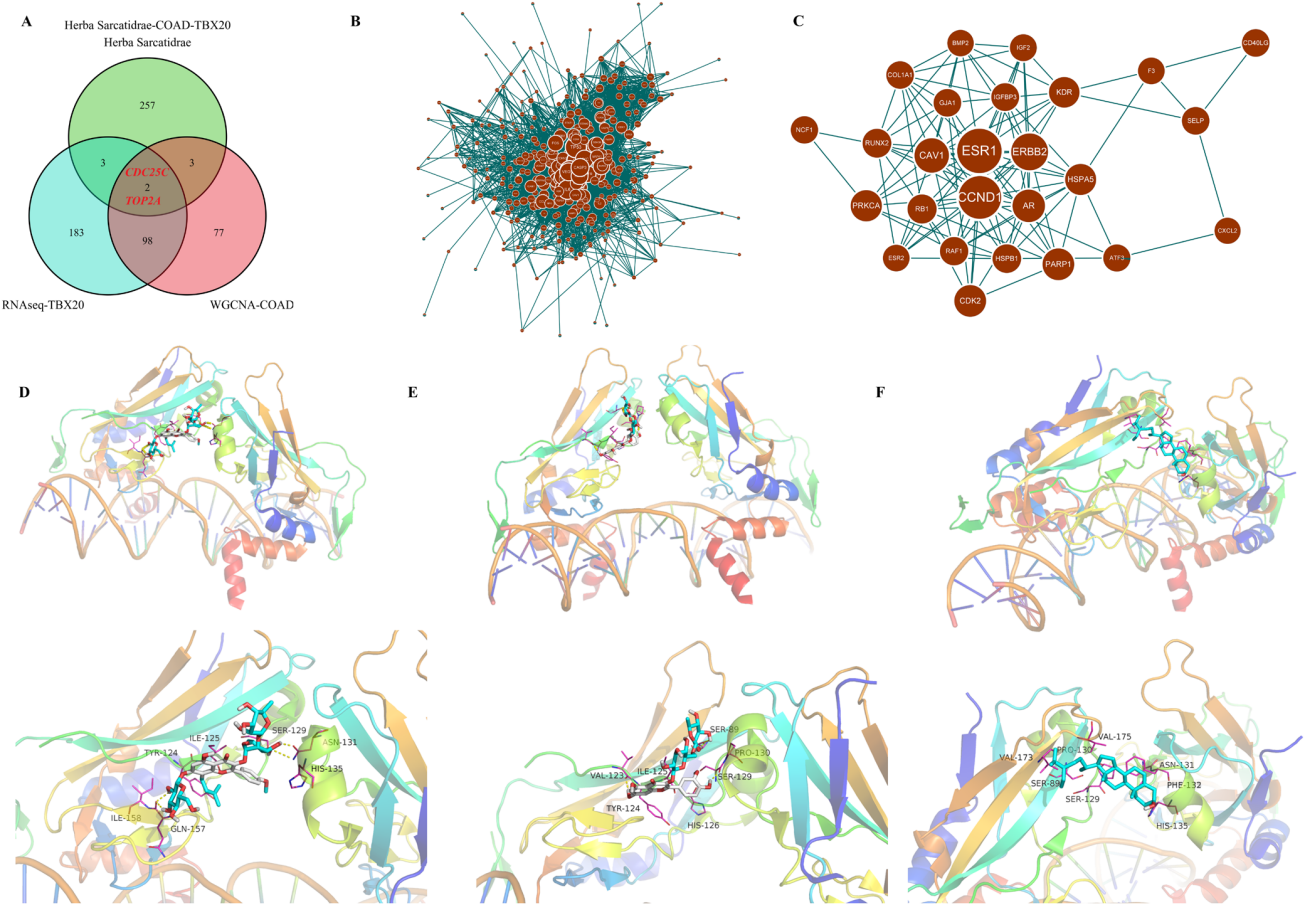
Correlation between the targets of active components in HS and the core target genes of TBX20. (A) Venn diagram of targets of HS active ingredients (*n* = 257), DEGs in TBX20 overexpressed CRC cells (*n* = 183), and co‐expressed genes obtained by WGCNA analysis (*n* = 77). (B) The target network of HS targets‐TBX20 overexpressed DEGs‐WGCNA was constructed using Cytoscape 3.7.2 software. (C) Twenty‐six potential core target genes for HS targets combined with TBX20 for the treatment of CRC patients were predicted using MCODE based on the Degree algorithm. The binding of TBX20 to Epimedin C (D), rutin (E), and β‐sitosterol (F) was analyzed by the molecular docking method.

Based on the analysis, the data found 257 targets of HS active ingredients, 183 DEGs in TBX20 overexpressed CRC cells, and 77 co‐expressed genes obtained by WGCNA analysis. Through the Venn diagram analysis, two potential targets (CDC25C and TOP2A) were obtained (Figure 2A). Subsequently, the target network of HS‐TBX20 overexpressing DEGs‐WGCNA was constructed by Cytoscape 3.7.2 software (Figure 2B). Through prediction with MCODE based on the Degree algorithm, we found that there were 26 potential core target genes of HS, which could be combined with TBX20 for the treatment of CRC patients (Figure 2C). The 41 active components of HS were subjected to molecular docking to identify the potential drug candidates targeting TBX20. The PDB structure of the core protein TBX20 was downloaded from the PDB data. The sdf format files of 41 HS active ingredients were downloaded from NCBI's PubChem database. All of these drugs were docked to the TBX20 core protein and ranked according to LibDockScore. TBX20 was found to be associated with Epimedin C, Engelitin, chloranoside a, rutin, lignoceric acid, Docosanoate, and pelargonidin‐3‐rhamnosyl glucoside, β‐sitosterol, and sitosterol, which may be the drugs targeting TBX20. The protein binding of TBX20 to Epimedin C, rutin, and β‐sitosterol was analyzed by the molecular docking method. Subsequently, the drugs were docked using AutoDock‐Vina for more accurate binding to the TBX20 structural domain. Epimedin C, Engelitin, chloranoside a, rutin, lignoceric acid, Docosanoate, pelargonidin‐3‐rhamnosyl glucoside, β‐sitosterol, and sitosterol were used as drug candidates targeting the active sites of TBX20. The results showed that the affinity of the drugs for TBX20 was −3.3 kcal‐mol‐1 or less. Combining LibDockScore, binding energy, and RMSD values, the data revealed that Epimedin C, rutin, and β‐sitosterol formed the best docking complex with TBX20. Then, the binding of TBX20 to Epimedin C, rutin, and β‐sitosterol was analyzed by the molecular docking method (Figure 2D–F).

### 3.4 β‐Sitosterol Suppresses Proliferation and Promotes Apoptosis of CRC Cells

**FIGURE 3:**
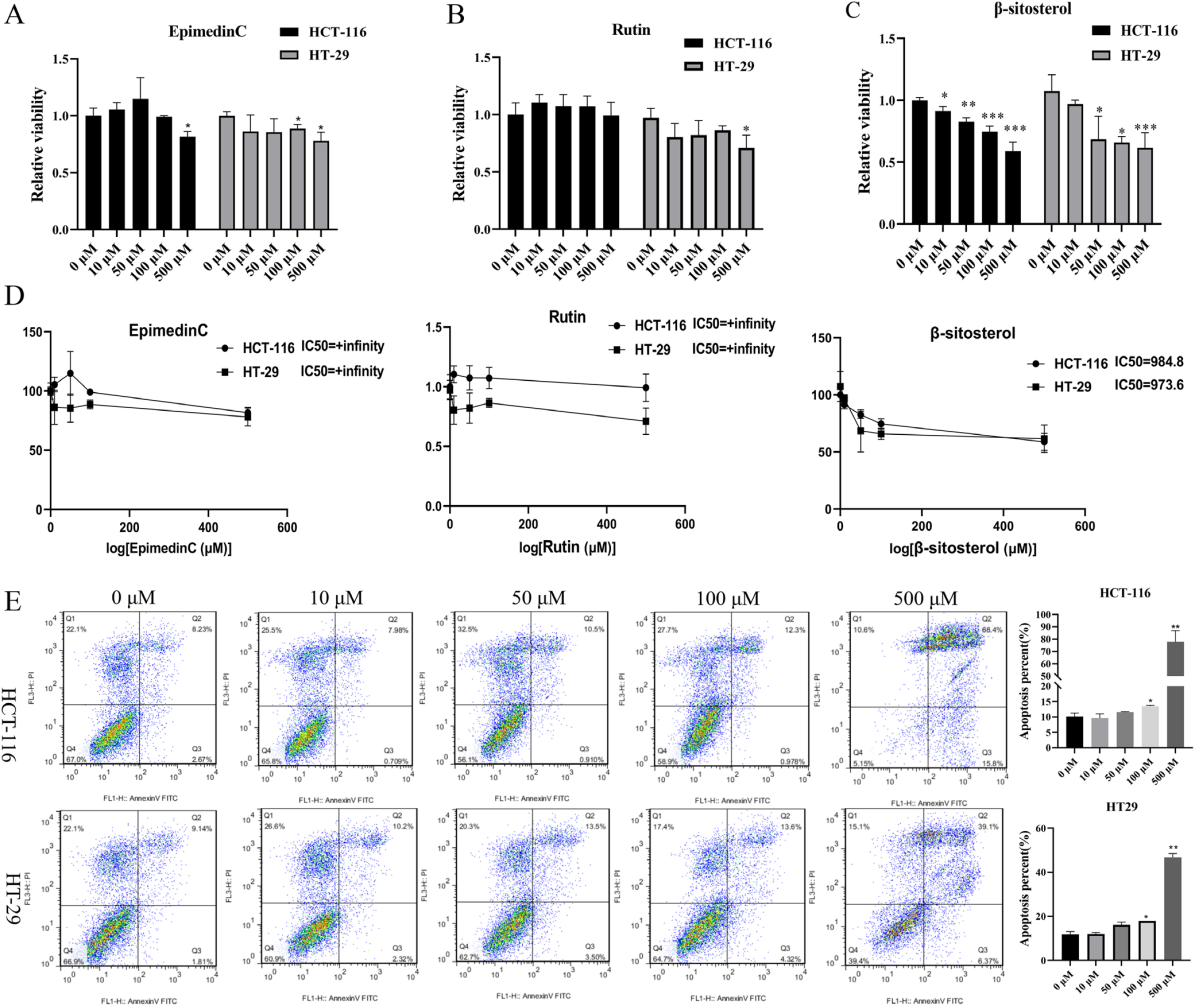
β‐sitosterol suppresses the proliferation and promotes apoptosis of CRC cells. HCT‐116 and HT‐29 cells were treated with 0, 10, 50, 100, and 500 μM Epimedin C, rutin, and β‐sitosterol, which were the top three active substances bound by TBX20 for 48 h, respectively. CCK‐8 was adopted to confirm the effects of Epimedin C (A), rutin (B), and β‐sitosterol (C) on the proliferation of CRC cells. (D) IC50 values of Epimedin C, rutin, and β‐sitosterol were determined in HCT‐116 and HT‐29 cells. (E) Flow cytometry was applied to the effect of different concentrations of β‐sitosterol on the apoptotic ability of HCT‐116 and HT‐29 cells. *p < 0.05, **p < 0.01, ***p < 0.001.

Next, Epimedin C, rutin, and β‐sitosterol were selected and applied in subsequent experiments, as they were the top three active substances predicted to bind TBX20. In this part, HCT‐116 and HT‐29 cells were treated with 0, 10, 50, 100, and 500 μM Epimedin C, rutin, and β‐sitosterol. CCK‐8 data signified that β‐sitosterol (10, 50, 100, and 500 μM) markedly inhibited the proliferation of HCT‐116 cells, and β‐sitosterol (50, 100, and 500 μM) inhibited the proliferation of HT‐29 cells. Epimedin C (only 500 μM) reduced the viability of HCT‐116 cells, and Epimedin C (100 and 500 μM) also showed inhibitory effects on HT‐29 cells. Rutin exhibited an inhibitory effect only at 500 μM on HT‐29 cells (Figure 3A–C). To compare the drug sensitivity of Epimedin C, rutin, and β‐sitosterol, IC50 values were determined in HCT‐116 and HT‐29 cells. The CCK‐8 results showed that β‐sitosterol had a stronger inhibitory effect compared to Epimedin C and rutin, which exhibited weaker effects on both cell lines (Figure 3D). These results revealed that β‐sitosterol exerted the strongest inhibitory effect on CRC cell proliferation and was thus selected for further study. Flow cytometry analysis further confirmed that β‐sitosterol (100 and 500 μM) notably enhanced apoptosis in both HCT‐116 and HT‐29 cells (Figure 3E).

### 3.5 HS and β‐Sitosterol Attenuate CRC Tumour Formation and Upregulate TBX20 in Nude Mice

**FIGURE 4:**
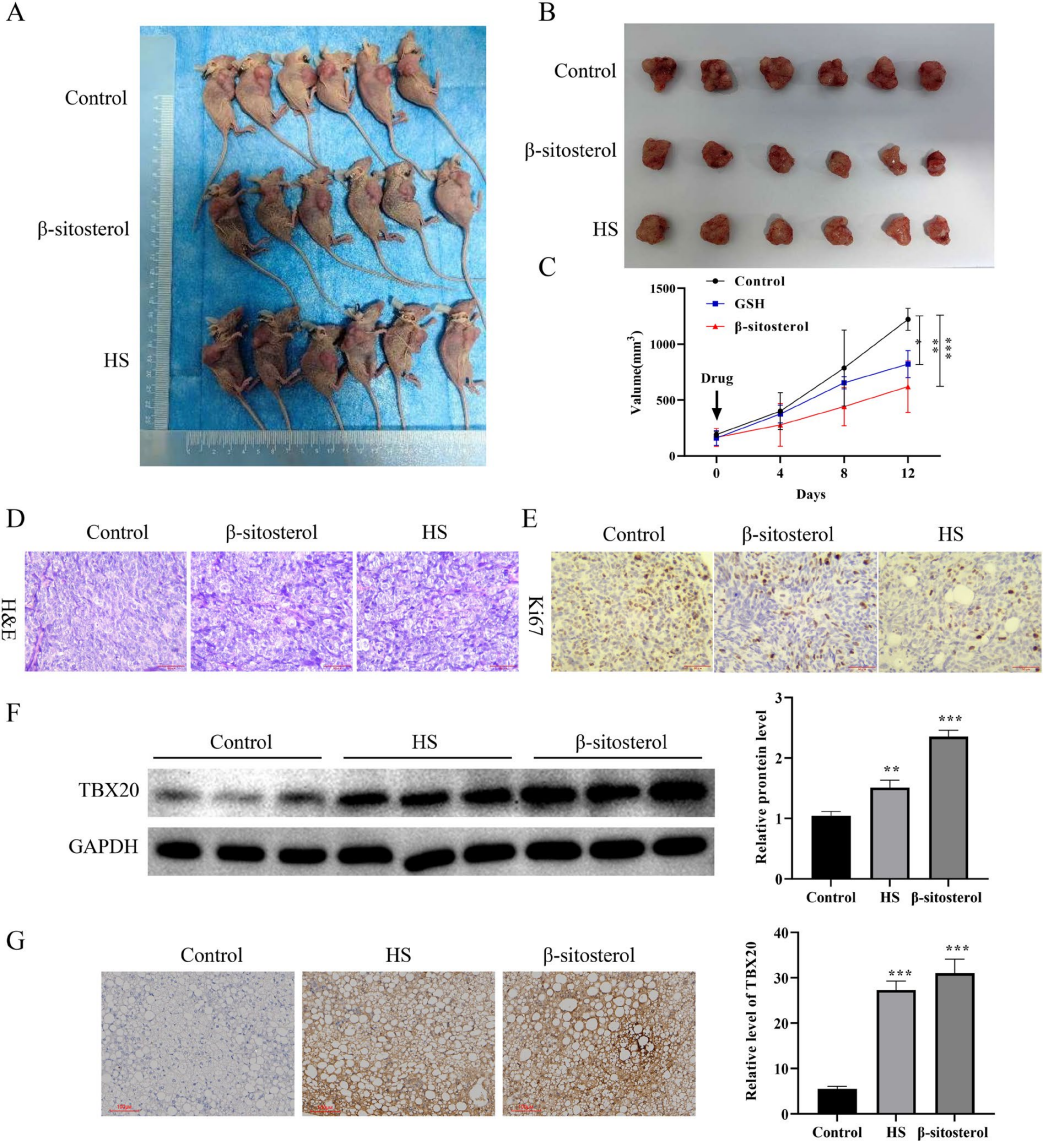
HS and β‐sitosterol inhibit CRC tumor formation and enhance TBX20 expression in nude mice. HT‐29 cells were subcutaneously injected into the armpit of nude mice. After tumor formation, the mice received daily treatment for 12 days with HS (0.2 mL by oral gavage) or β‐sitosterol (0.2 mL by intraperitoneal injection). (A) The nude mice were killed, photographed, and exhibited at the end of the experiment. (B) The groups of tumors were displayed in nude mice by photographing. (C) Tumor length and width were measured, and volume was calculated at 0, 4, 8, and 12 days after drug treatment. (D) The pathological structure of the tumor was assessed using H&E staining. (E) The expression of Ki67 in the tumors was evaluated by IHC assay. (F) Western blotting analysis of TBX20 protein expression in tumor tissues. (G) IHC staining of TBX20 expression in tumor sections. *p < 0.05, **p < 0.01, ***p < 0.001.

The results uncovered that HS and β‐sitosterol had significant inhibitory effects on the tumorigenesis of HCT‐29 cells (Figure 4A–C). H&E staining results exhibited that in the control group, the cells were swollen and enlarged, with obvious heterogeneity and localized inflammatory cell infiltration; in the HS and β‐sitosterol treatment group, tissue heterogeneity was not obvious, vascular hyperplasia was scarce, and tumor cells were small in size, especially in the β‐sitosterol treatment group (Figure 4D). IHC results displayed that the level of Ki67 was prominently reduced in the HS and β‐sitosterol treatment group, especially in the β‐sitosterol treatment group (Figure 4E). Moreover, Western blot and IHC analyses revealed that both HS and β‐sitosterol treatments notably increased the protein expression of TBX20 relative to the control group, and the increase in TBX20 expression was more pronounced in the β‐sitosterol‐treated group than in the HS group (Figure 4F,G).

### 3.6 β‐Sitosterol Enhances the Sensitivity of CRC to 5‐FU and Oxaliplatin

**FIGURE 5:**
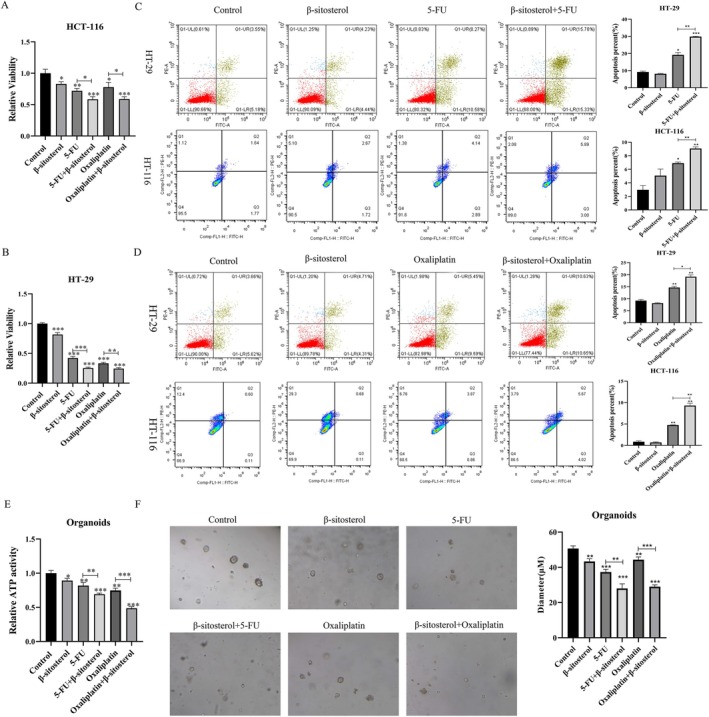
β‐sitosterol enhances the sensitivity of CRC to 5‐FU and oxaliplatin. HCT‐116 and HT‐29 cells were treated with 50 μM β‐sitosterol, 5 μg/mL 5‐FU, and 5 μg/mL Oxaliplatin for 48 h. CCK‐8 monitored cell viability in HCT‐116 (A) and HT‐29 cells (B). (C) Apoptosis assay by flow cytometry in HCT‐116 and HT‐29 cells treated with β‐sitosterol and 5‐FU for 48 h. (D) Apoptosis assay by flow cytometry in HCT‐116 and HT‐29 cells treated with β‐sitosterol and Oxaliplatin for 48 h. CRC organoids were constructed and treated with 50 μM β‐sitosterol, 5 μg/mL 5‐FU, 5 μg/mL Oxaliplatin, 50 μM β‐sitosterol plus 5 μg/mL 5‐FU, and 50 μM β‐sitosterol plus 5 μg/mL Oxaliplatin for 48 h. (E) ATP activity was monitored using a kit in each group of CRC organoids. (F) The organoids were photographed and displayed, and the diameter was measured. *p < 0.05, **p < 0.01, ***p < 0.001.

Next, CCK‐8 results manifested that β‐sitosterol, 5‐FU, and Oxaliplatin reduced the viability of HCT‐116 and HT‐29 cells; the combined treatments of β‐sitosterol and 5‐FU or β‐sitosterol and Oxaliplatin had more significant inhibitory effects on the viability of HCT‐116 and HT‐29 cells than 5‐FU or Oxaliplatin alone treatment group (Figure 5A,B). Besides, the data revealed that the combined treatment of β‐sitosterol and 5‐FU had a stronger induction effect on the apoptosis of HCT‐116 and HT‐29 cells than the 5‐FU alone treatment group (Figure 5C). Similarly, the combined treatment of β‐sitosterol and Oxaliplatin also markedly enhanced the apoptosis of HCT‐116 and HT‐29 cells relative to the Oxaliplatin alone treatment group (Figure 5D). Additionally, β‐sitosterol, 5‐FU, and Oxaliplatin decreased the ATP activity of CRC organoids compared with the control group; the combined treatments of β‐sitosterol and 5‐FU or β‐sitosterol and Oxaliplatin further reduced the ATP activity of CRC organoids relative to the 5‐FU or Oxaliplatin alone treatment group (Figure 5E). Meanwhile, β‐sitosterol, 5‐FU, and Oxaliplatin alone could inhibit the growth of CRC organoids, and the combined treatment had a better inhibitory effect (Figure 5F).

### 3.7 β‐Sitosterol Prevents CRC Progression by Enhancing TBX20 Protein Stability

**FIGURE 6:**
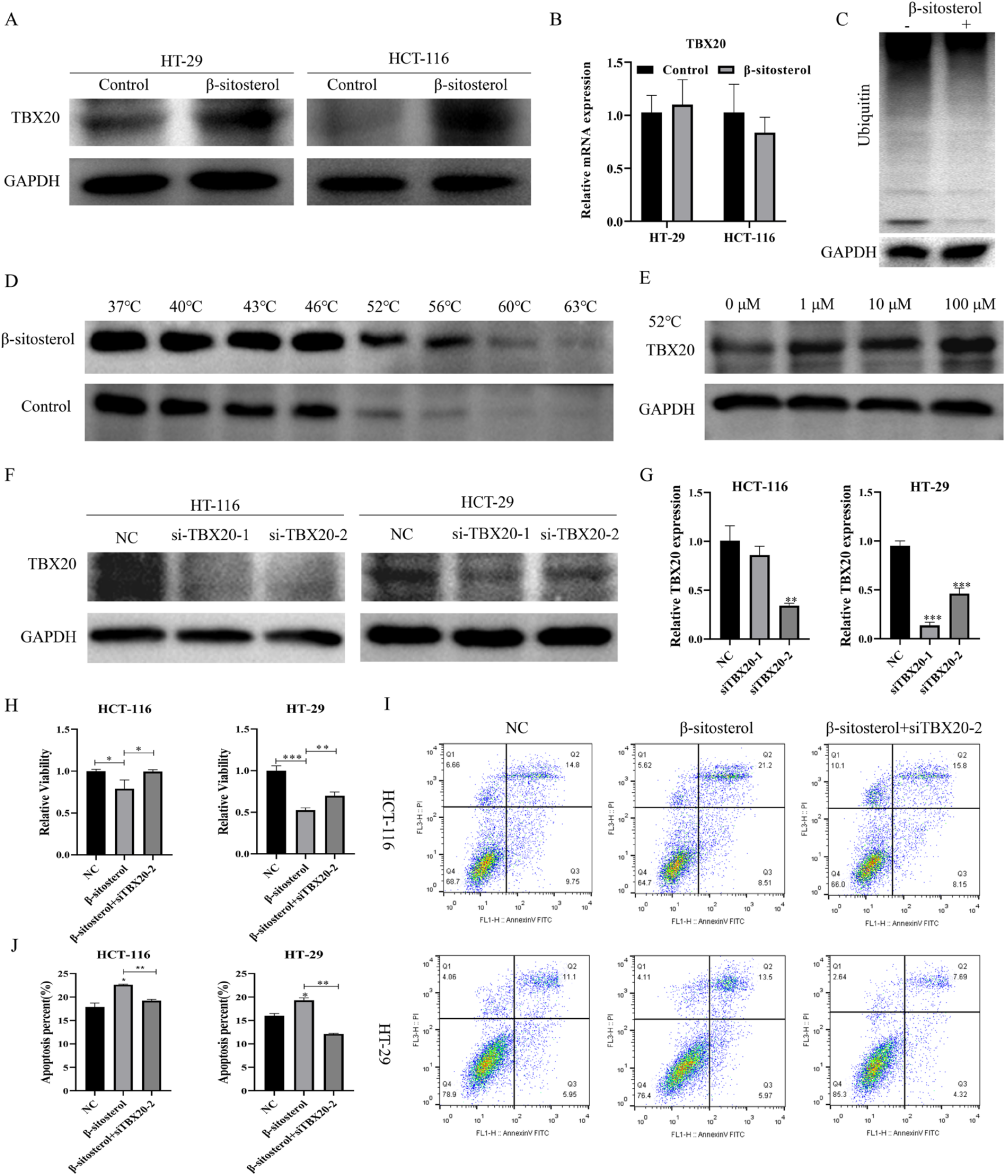
β‐sitosterol prevents CRC progression by enhancing TBX20 protein stability. HCT‐116 and HT‐29 cells were processed with 100 μM β‐sitosterol for 48 h. (A) The expression of TBX20 was examined by Western blot. (B) QRT‐PCR assessed the mRNA level of TBX20. (C) The effect of β‐sitosterol on TBX20 ubiquitination was investigated by CO‐IP before and after β‐sitosterol treatment. (D) The relative stability of proteins at 37°C, 40°C, 43°C, 46°C, 52°C, 56°C, 60°C, and 63°C was examined using protein thermal shift technology in HCT‐116 cells treated with 100 μM β‐sitosterol. (E) The stability of TBX20 was determined using protein thermal shift technology in HCT‐116 cells treated with different concentrations of β‐sitosterol at 52°C. (F) HCT‐116 and HT‐29 cells were transfected with TBX20 siRNA‐1 and siRNA‐2. The TBX20 protein was detected by Western blot. (G) Quantification of TBX20 protein. (H) TBX20‐silenced HCT‐116 and HT‐29 cells were treated with 100 μM β‐sitosterol for 48 h, and cell proliferation was assessed by CCK‐8. (I) A flow cytometer was applied to evaluate the effect of β‐sitosterol and TBX20 silencing on the apoptosis of HCT‐116 and HT‐29 cells. (J) The apoptosis rate was calculated according to the results of flow cytometry. *p < 0.05, **p < 0.01, ***p < 0.001.

More importantly, this study investigated whether β‐sitosterol could affect CRC proliferation and apoptosis by regulating TBX20 protein stability. Western blotting results denoted that 100 μM β‐sitosterol increased the protein level of TBX20, but had no effect on its mRNA level in HCT‐116 and HT‐29 cells (Figure 6A,B). CO‐IP results further showed that treatment with β‐sitosterol resulted in a significant decrease in the ubiquitination of TBX20, suggesting that β‐sitosterol might stabilize TBX20 by inhibiting its ubiquitin‐mediated degradation (Figure 6C). Then, the data of protein thermal shift technology signified that after treatment with 100 μM β‐sitosterol, HCT‐116 cell proteins were relatively stable at 52°C–56°C (Figure 6D). Besides, the stability of TBX20 was increased after treatment of HCT‐116 protein with a concentration gradient of β‐sitosterol at 52°C in a concentration‐gradient manner (Figure 6E). Next, HCT‐116 and HT‐29 cells were transfected with TBX20 siRNA‐1 and siRNA‐2. Western blot data uncovered that TBX20 silencing downregulated TBX20 in HCT‐116 and HT‐29 cells, especially transfection of TBX20 siRNA‐2 (Figure 6F,G). Functionally, CCK‐8 results exhibited that TBX20 knockdown reversed the inhibition of cell proliferation mediated by β‐sitosterol in HCT‐116 and HT‐29 cells (Figure 6H). Flow cytometry results manifested that TBX20 knockdown attenuated the increase in apoptosis induced by β‐sitosterol in HCT‐116 and HT‐29 cells (Figure 6I,J).

## 4 Discussion

CRC is a common digestive tract tumor with high mortality (about 935,000 deaths) worldwide [19]. CRC patients are mainly middle‐aged and elderly people [20], and most of the lesions are located in intestinal mucosal epithelial cells [21]. The early symptoms of CRC are not obvious and are easy to ignore. Most patients are diagnosed at an advanced stage [22]. Surgical resection is the mainstay of treatment for CRC, but the recurrence rate is high [23]. Adjuvant chemotherapy is beneficial after surgery. However, chemotherapy can produce a series of adverse reactions [24]. TCM, as an alternative and complementary therapeutic agent, has been widely applied in the TCM clinical treatment of cancer [25, 26]. Studies confirmed that TCM can reduce the side effects of conventional treatment [27, 28]. Therefore, finding an effective adjuvant TCM for the treatment of CRC is urgently needed in clinical practice. Clinical studies demonstrated that HS has a good anti‐tumor effect, and combined with chemotherapy can play the role of improving efficiency and depressing toxicity [1, 3, 4]. It has been found that HS has cytotoxic and tumor‐suppressive effects on multiple tumors, such as gastrointestinal tumors, respiratory tumors, and breast cancer. However, the therapeutic effect and mechanism of HS on CRC remain unclear.

In this study, a network pharmacology approach was applied to reveal the “multi‐component, multi‐target, and multi‐pathway” mechanism of HS in CRC therapy. Through multiple drug‐related databases, 41 HS active components and 265 potential targets were obtained. DO analysis found that a total of 48 HS component target genes were enriched in CRC diseases, including PTGS2, DPP4, MAPK14, GSK3B, ACHE, CCND1, TNF, BAX, BCL2, CASP3, CASP8, etc. GO analysis showed that the target genes of HS components were mainly enriched in xenobiotic stimulus and lipopolysaccharide reaction. KEGG analysis showed that the target genes of HS components were mainly enriched in the AGE‐RAGE pathway in diabetic complications, Lipid, and atherosclerosis.

TBX20, as one of the crucial members of the T‐box transcription factor family, plays a key role in the development of the heart and branchial arches [9]. Previous and our unpublished study disclosed that TBX20 has a crucial role in CRC progression [11]. Through TCGA database analysis, 206 TBX20‐related DEGs were obtained. Among them, several genes have significant effects on the survival prognosis and TMN stage of COAD, such as NTN5, PPP1R1A, EFHD1, EME1, GLDN, CYP4F12, C1R1.AS1, VWCE, TRPM5, and TLR6. Besides, two potential targets (CDC25C and TOP2A) were identified by analyzing the intersection of 265 HS active component targets, 286 TBX20‐related DEGs, and 180 co‐expressed genes obtained by WGCNA analysis.

In this study, it was also found that the active components of HS (Epimedin C, rutin, and β‐sitosterol) formed a good docking complex with the core protein TBX20. Our results denoted that β‐sitosterol had the strongest inhibitory effect on the proliferation of CRC cells relative to Epimedin C and rutin. β‐sitosterol had a significant pro‐apoptotic effect on CRC cells. β‐sitosterol is a major component of cell membranes in animals and plants, which can reduce blood lipids and inhibit lipid peroxidation [29]. β‐sitosterol also has anti‐inflammatory activity and has certain therapeutic effects on pancreatic cancer [30], liver cancer [31], and cervical cancer [32]. This study further confirmed through in vivo experiments that HS and β‐sitosterol can inhibit the tumorigenesis of CRC cells and increase TBX20 protein levels in nude mice, and the inhibitory effect of β‐sitosterol is stronger than that of HS.

Moreover, it has been shown that β‐sitosterol can suppress CRC multidrug resistance by attenuating p53 and MDM2 interactions [33]. 5‐FU and oxaliplatin, as the first‐line chemotherapeutic agents for CRC, have an overall effective rate of 25%‐50% [34]. Therefore, it is of great significance to explore whether β‐sitosterol can reduce the resistance of CRC to 5‐FU and oxaliplatin for treating CRC. In this study, our data revealed that β‐sitosterol, 5‐FU, and oxaliplatin alone could inhibit proliferation and enhance apoptosis in HCT‐116 and HT‐29 cells; β‐sitosterol, 5‐FU, and oxaliplatin also could decrease ATP activity and suppress the growth of CRC organoids; the combined treatment had a stronger inhibitory effect than the individual treatment in CRC cells and organoids.

At present, the regulation of TBX20 by β‐sitosterol has not been reported. This study is the first to confirm that β‐sitosterol upregulated TBX20 protein and increased the stability of TBX20. Besides, functional experiments proved that β‐sitosterol could prevent proliferation and induce apoptosis of CRC cells by enhancing TBX20 protein stability. Further, β‐sitosterol might stabilize TBX20 by inhibiting its ubiquitin‐mediated degradation. Nevertheless, it should be noted that the predicted binding between β‐sitosterol and TBX20 was based solely on molecular docking simulations, and direct experimental validation of this interaction is still lacking. This remains a limitation of the current study. We will further verify the molecular target of β‐sitosterol through experiments in future study.

## 5 Conclusions

This study identified a range of HS active ingredient target genes and TBX20‐related genes through network pharmacology and TCGA analysis. Molecular docking revealed that β‐sitosterol exhibited the strongest inhibitory effect on CRC. Additionally, β‐sitosterol was shown to enhance TBX20 protein stability and increase CRC sensitivity to chemotherapy. Thus, HS and β‐sitosterol may serve as effective adjuncts to conventional chemotherapy for CRC treatment.

## Author Contributions

**Haixiao Yuan:** conceptualization (equal), data curation (equal), formal analysis (equal), investigation (equal), methodology (equal), writing – original draft (equal), writing – review and editing (equal). **Weiqing Feng:** conceptualization (equal), data curation (equal), formal analysis (equal), investigation (equal), methodology (equal), writing – original draft (equal), writing – review and editing (equal). **Shaohua Yang:** formal analysis (equal), investigation (equal), methodology (equal), writing – review and editing (equal). **Hao Yin:** formal analysis (equal), investigation (equal), methodology (equal), writing – review and editing (equal). **Shaoyong Ouyang:** formal analysis (equal), investigation (equal), methodology (equal), writing – review and editing (equal). **Hong Xie:** formal analysis (equal), investigation (equal), methodology (equal), writing – review and editing (equal). **Hongmei Tang:** formal analysis (equal), investigation (equal), methodology (equal), writing – review and editing (equal). **Xiaowei Ou:** formal analysis (equal), investigation (equal), methodology (equal), writing – review and editing (equal). **Xianling Gong:** formal analysis (equal), investigation (equal), methodology (equal), writing – review and editing (equal). **Jie Yuan:** conceptualization (lead), data curation (equal), formal analysis (equal), investigation (equal), methodology (equal), writing – review and editing (equal).

## Ethics Statement

All animal experiments were approved by the Ethical Committee of Guangzhou Forevergen Medical Laboratory Animal Center (approval no: IACUC‐AEWC‐F2211008). All animal studies should also comply with the ARRIVE guidelines and the AVMA euthanasia guidelines 2020.

## Consent

The authors have nothing to report.

## Conflicts of Interest

The authors declare no conflicts of interest.

## Supporting information


**Data S1.** supporting Information.

## Data Availability

The data generated in the present study are included in the figures and tables of this article.
