# Supplementary material for: β‐Sitosterol as an Anti‐Tumour Active Component of Herba Sarcandrae Inhibits Colorectal Cancer Progression Through Up‐Regulation of TBX20
Source: J Cell Mol Med. 2025 Sep 1;29(17):e70809. doi: 10.1111/jcmm.70809 (PMC12401127; doi:10.1111/jcmm.70809)
Supplement: Supplementary file 1 — Data S1. supporting Information. [file JCMM-29-e70809-s001.docx]

**
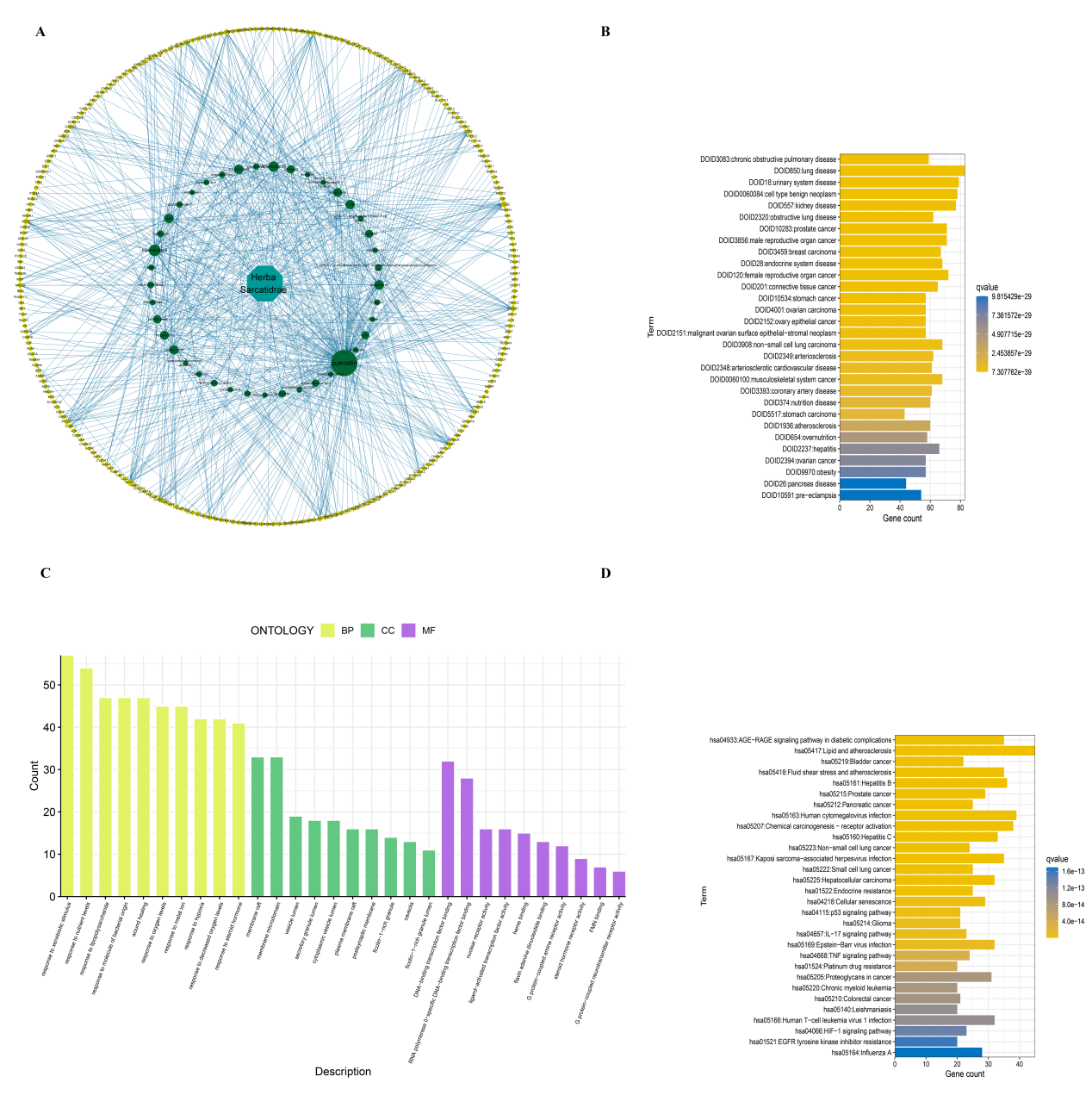
Figure S1. Cyberpharmacological analysis of the components and targets of HS.** (A) The ingredient-target relationship was obtained through multiple drug-related databases including SymMap, TCMID, TCMSP, and TCM-ID. (B) DO analysis of potential targets of HS. (C) GO analysis of potential targets of HS. (D) KEGG analysis of potential targets of HS.

**
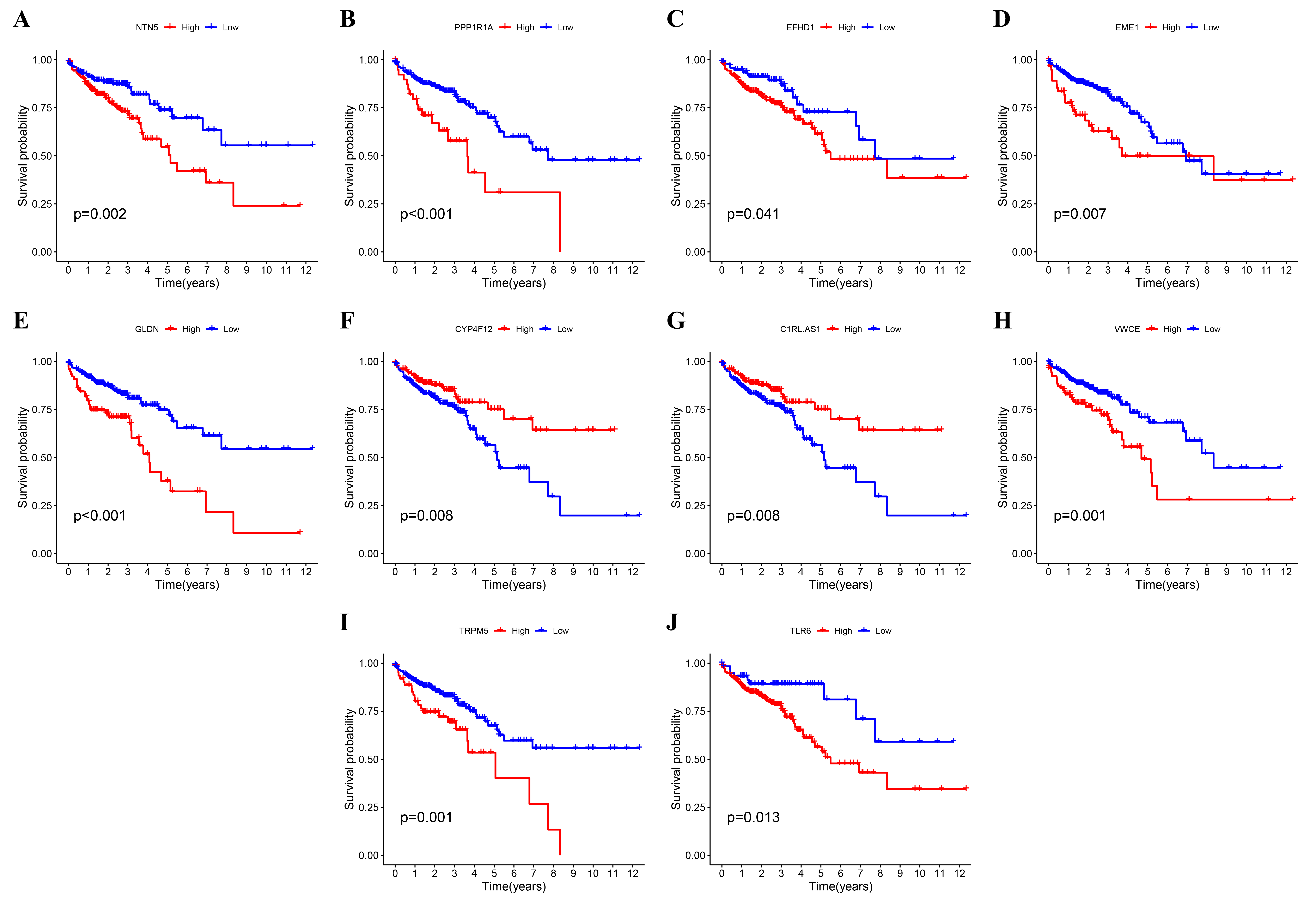
Figure S2. Survival prognosis in COAD.** NTN5 (A), PPP1R1A (B), EFHD1 (C), EME1 (D), GLDN (E), CYP4F12 (F), C1RL.AS1 (G), VWCE (H), TRPM5 (I), and TLR6 (J) expression had a significant effect on survival prognosis in COAD


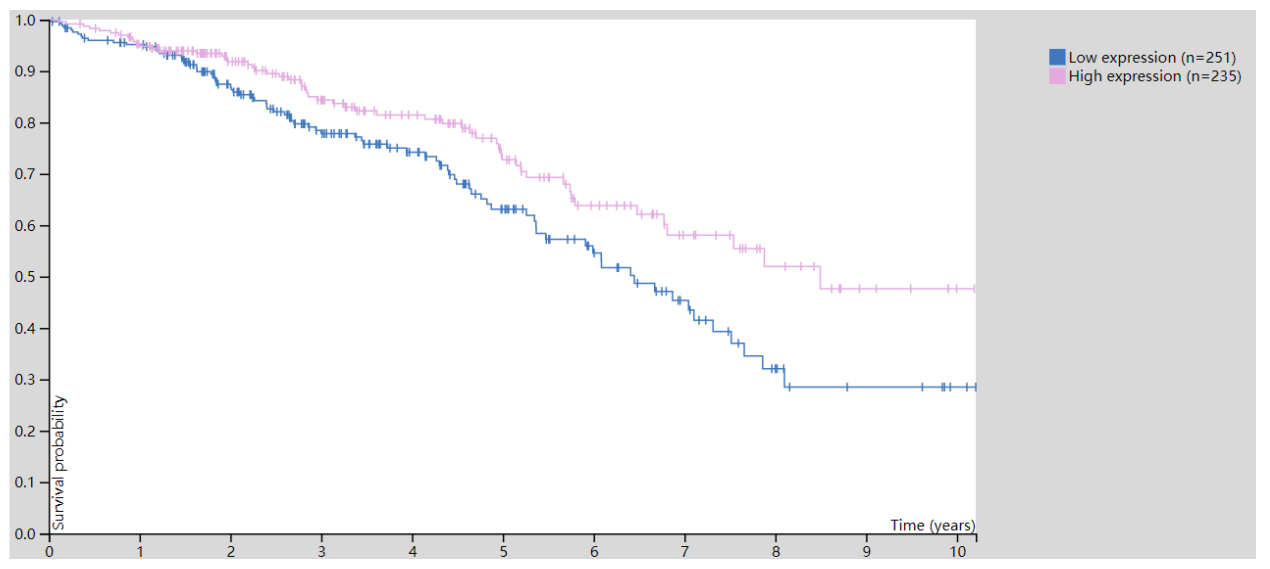


**Figure S3. Kaplan-Meier survival analysis of TBX20 expression in COAD.** The analysis was based on the TCGA database and stratified by TBX20 expression levels.

**Supplementary Table S1**. Raw molecular docking results between HS active components and TBX20 protein. Docking was performed using AutoDock Vina.
